# High Pulmonary Levels of IL-6 and IL-1β in Children with Chronic Suppurative Lung Disease Are Associated with Low Systemic IFN-γ Production in Response to Non-Typeable *Haemophilus influenzae*

**DOI:** 10.1371/journal.pone.0129517

**Published:** 2015-06-12

**Authors:** Susan J. Pizzutto, John W. Upham, Stephanie T. Yerkovich, Anne B. Chang

**Affiliations:** 1 Child Health Division, Menzies School of Health Research, Charles Darwin University, Casuarina, NT, Australia; 2 Department of Respiratory Medicine, Princess Alexandra Hospital, Woolloongabba, QLD, Australia; 3 School of Medicine, The University of Queensland, Brisbane, QLD, Australia; 4 Queensland Lung Transplant Service, The Prince Charles Hospital, Brisbane, QLD, Australia; 5 Department of Respiratory and Sleep Medicine, Children’s Health Queensland and Queensland Children’s Medical Research Institute, Queensland University of Technology, Brisbane, QLD, Australia; Imperial College London, UNITED KINGDOM

## Abstract

Non-typeable *Haemophilus influenzae (NTHi)* is commonly associated with chronic suppurative lung disease in children. We have previously shown that children with chronic suppurative lung disease have a reduced capacity to produce IFN-γ in response to *NTHi* compared with healthy control children. The aim of this study was to determine if deficient NTHi-specific IFN-γ production is associated with heightened systemic or airway inflammation. We measured a panel of cytokines (IFN-γ, IL-1β, IL-6, IL-8, IL-12 p70), antimicrobial proteins (LL-37, IP-10) as well as cellular and clinical factors associated with airway and systemic inflammation in 70 children with chronic suppurative lung disease. IFN-γ was measured in peripheral blood mononuclear cells challenged *in vitro* with live NTHi. Regression analysis was used to assess the association between the systemic and airway inflammation and the capacity to produce IFN-γ. On multivariate regression, NTHi-specific IFN-γ production was significantly negatively associated with the BAL concentrations of the inflammatory cytokines IL-6 (β=-0.316; 95%CI -0.49, -0.14; p=0.001) and IL-1β (β=-0.023; 95%CI -0.04, -0.01; p=0.001). This association was independent of bacterial or viral infection, BAL cellularity and the severity of bronchiectasis (using modified Bhalla score on chest CT scans). We found limited evidence of systemic inflammation in children with chronic suppurative lung disease. In summary, increased local airway inflammation is associated with a poorer systemic cell-mediated immune response to NTHi in children with chronic suppurative lung disease. These data support the emerging body of evidence that impaired cell-mediated immune responses and dysregulated airway inflammation may be linked and contribute to the pathobiology of chronic suppurative lung disease.

## Introduction

Chronic suppurative lung disease (CSLD) is commonly encountered in paediatric respiratory clinics globally. CSLD is characterised by persistent wet cough and recurrent lower respiratory infections and neutrophilic inflammation in the airways. CSLD shares many clinical characteristics with and is thought to be a common antecedent of bronchiectasis [1]. Thus studying CSLD in young children may provide important insight into the pathogenesis of bronchiectasis.

Non-typeable *Haemophilus influenzae* (NTHi) is a common and important respiratory pathogen associated with CSLD/bronchiectasis [2–4]. Accumulating evidence from studies in adults [5,6] and children [7,8] suggest that a low capacity for IFN-γ production in response to NTHi may contribute to the cycle of infection and inflammation associated with the pathogenesis of bronchiectasis. Understanding the mechanisms that contribute to impaired immune responses and the pathogenesis of CSLD are important for the development of intervention therapies. The mechanisms leading to the impaired NTHi-driven IFN-γ response described in children and adults have not been investigated. The innate immune response helps drive the adaptive immune response (e.g. NTHi-driven IFN-γ response) however, our previous work showed limited evidence of impaired innate immune responses to NTHi in children with CSLD/bronchiectasis [8]. Thus, the current study focuses on possible downstream factors associated with impaired systemic NTHi-driven IFN-γ responses.

There is a growing body of evidence that persistent airway or systemic inflammation may contribute to impaired T-cell responses. Severe acute inflammation and chronic infection can disrupt macrophage and T-cell activation and subsequently induce an environment of immune tolerance or exhaustion [9,10]. Furthermore, it is increasingly recognised that lung and systemic immune responses may be inter-related [11,12]. Given these associations, in this prospective study involving 70 young children with CSLD/bronchiectasis, we investigated whether inflammation (local and systemic) is related to the capacity to produce IFN-γ in response to NTHi. We hypothesised that an impaired systemic ability to produce IFN-γ in response to NTHi is associated with lung or systemic inflammation. The most notable finding from this study was that NTHi-specific IFN-γ production by blood mononuclear cells was associated with inflammation.

## Materials and Method

### Study participants

Children ≤ 10 years of age with CSLD, undergoing chest high resolution computed tomography (HRCT) and flexible bronchoscopy for suspected bronchiectasis, were prospectively recruited (2010–2013) from the Royal Darwin Hospital, Northern Territory, Australia. All children were clinically stable (defined as absence of recent exacerbation) and under the care of a specialist paediatrician at the time of sample collection. Radiographic diagnosis of bronchiectasis was made by the respiratory paediatrician (AC) and the severity of bronchiectasis scored using a modified Bhalla scale as previously done [13]. The total score was the sum of the score for each lobe, including lingula, based on the extent of bronchiectasis, bronchial wall thickness and dilation (maximum score 48). Routine clinical investigations [14] were undertaken in all children evaluated for bronchiectasis. Clinical and socio-demographic data were collected using standardised data collection forms. Blood and bronchoalveolar lavage (BAL) for clinical and research investigations were collected immediately prior to chest CT scan/bronchoscopy as previously described [8]. This study was approved by the Human Research Ethics Committee (Northern Territory Department of Health and Menzies School of Health Research; #07/63) and children enrolled following written informed consent from the parent/carer.

### Sample collection

Peripheral blood mononuclear cells (PBMC) and plasma were collected and stored as previously described [8]. Briefly, PBMC were isolated from heparinised blood using standard density-gradient techniques and cryopreserved in liquid nitrogen until use. Matched plasma was stored at -80°C.

BAL was collected and processed as previously described [15]. Briefly, BAL was collected as two separate aliquots from the most affected lobe and maintained on ice for up to 3 hours prior to processing. The first aliquot was used for microbiologic/viral analysis, the second for cytology and immunology.

BAL used for bacterial culture was stored diluted 2 fold in skim milk tryptone glucose glycerine broth at -80°C. BAL for viral or bacterial analysis by PCR was stored undiluted at -80°C. Samples were maintained at -80°C prior to testing.

### Microbiology/virology Investigations

Semi-quantitative culture identification of *Haemophilus influenzae*, *Streptococcus pneumoniae* and *Moraxella catarrhalis* from BAL were performed by our research laboratory and a threshold of >10^4^ CFU/ml considered clinically important [3,16]. *Pseudomonas aeruginosa* and *Klebsiella pneumoniae* culture and identification were performed by the diagnostic laboratory at the Royal Darwin Hospital. Presence of *Chlamydia pneumoniae*, *Mycobacteria pneumoniae* and the respiratory viruses (rhinovirus, adenovirus, enterovirus, bocavirus, respiratory syncytial virus, Wu virus, Ki virus, coronavirus, parainfluenzae and metapneumovirus) were determined by PCR by the Queensland Paediatric Infectious Disease Laboratory as previously done [17].

### PBMC investigations

PBMC were challenged with a single strain of NTHi as previously described [8]. Briefly, PBMC were cultured with live NTHi, PHA (positive control) or medium alone (base line control) for 72 hours, as a reflection of memory T cell function and IFN-γ measured in the harvested supernatant.

### Quantification of systemic and airway Inflammatory markers

Routine clinical investigations (eg full blood count including white cell count, platelets, C-reactive protein (CRP) and serum protein) [1] were performed using the regional reference laboratory Royal Darwin Hospital.

IFN-γ, IL-1β, IL-6, IL-8, IL-12 p70, LL-37and IP-10 in plasma and BAL, and IFN-γ and in PBMC culture supernatants were measured in our research laboratory using an in-house dissociation-enhanced lanthanide fluorescent immunoassay (DELFIA) as described previously [7,18]. Quantitative standard curves were generated from serial dilutions of recombinant human proteins and included on each plate. The limit of detection was 10 pg/ml. The cathelicidin LL-37 was measured in plasma and BAL using a high sensitivity ELISA kit (Hycult Biotech, The Netherlands). The limit of detection was 0.14 ng/ml. For analysis purposes, a value of 1/10 the limit of detection was assigned if the marker concentration was below the limit of detection.

### Data analyses

Data were analysed using the statistics package STATA 13 (StataCorp, USA). As the data did not follow a normal distribution, group data was described as median with interquartile range (IQR). Differences between groups were assessed using the Mann-Whitney U test. A two-tailed p-value ≤ 0.05 was considered significant. Univariate regression analysis was used to assess the potential of systemic and airway inflammatory markers and clinical factors to predict in vitro NTHi-specific IFN-γ production. NTHi-specific IFN-γ concentration was natural log transformed prior to regression analysis. Variables with a p value <0.1 were included in a multivariate regression model. Within this multivariate model, a p value of <0.05 was considered an independent predictor of the capacity to produce IFN-γ in vitro in response to NTHi.

## Results

### Characteristics of the children in the cohort

The majority of the 70 children in this study (Table 1) were young, with 84% of the children aged < 48 months. All but 3 children had radiographic evidence of bronchiectasis and of these, 68% had 3 or more lobes affected. The aetiology of bronchiectasis was presumed to be post-infectious as more than 80% of the children had been hospitalised at least once and 50% hospitalised at least twice for a lower respiratory infection. None of the children had primary immune deficiencies.

**Table 1:** Demographic characteristics and respiratory history of the cohort.

Variable	N = 70
Age, months, median (IQR)	27 (19–40)
Male, n (%)	39 (55.7)
Indigenous, n (%)	65 (92.9)
Gestational age (n = 66), weeks; median (IQR)	38 (35–40)
Bronchiectasis, number of children (%)	68 (95.8)
number of lobes, median (IQR)	3 (2–4)
^§^Severity score, median (IQR)	7 (5–10)
Chronic suppurative otitis media, n (%)	9 (12.9)
History of hospitalisation (respiratory), n (%)	59 (84.3)
Number of hospitalisations, median (IQR)	2 (1–3)
Age at first hospitalisation, months, median (IQR)	6 (3–9)
Respiratory vaccinations up to date, n (%)	70 (100)
Passive cigarette smoke exposure (n = 52), n (%)	42 (80.8)
Family history asthma (n = 48), n (%)	23 (47.9)

^§^ scored according to a modified Bhalla scale as previously reported [13].

Airway neutrophilia (>15%) was found in 41% of children. A clinically important level of bacterial infection (>10^4^ cfu/ml BAL) was present in 20 children (28%) and one or more viral pathogens detected in 30 (44%). Non-typeable *H*. *influenzae* (15.5%), *S*. *pneumoniae* (15.5%) and rhinovirus (29.4%) were the most common pathogens identified (detailed microbiology is presented in S1 Table).

BAL neutrophil % was not significantly associated with the presence of any bacteria (β = 0.19, 95%CI -12.8–13.2; p = 0.98,) or virus (β = 11.44, 95%CI -0.335–23.2; p = 0.057). However, BAL eosinophil % was significantly and positively associated with viral infection (β = 2.534, 95%CI 0.414–4.654; p = 0.020). BAL IL-1β, IL-6, IL-8 and IP-10 significantly correlated with BAL % neutrophils but not with BAL % eosinophils (S2 Table).

### Relationship of airway and systemic profiles with NTHi-specific IFN-γ production

**Fig 1:**
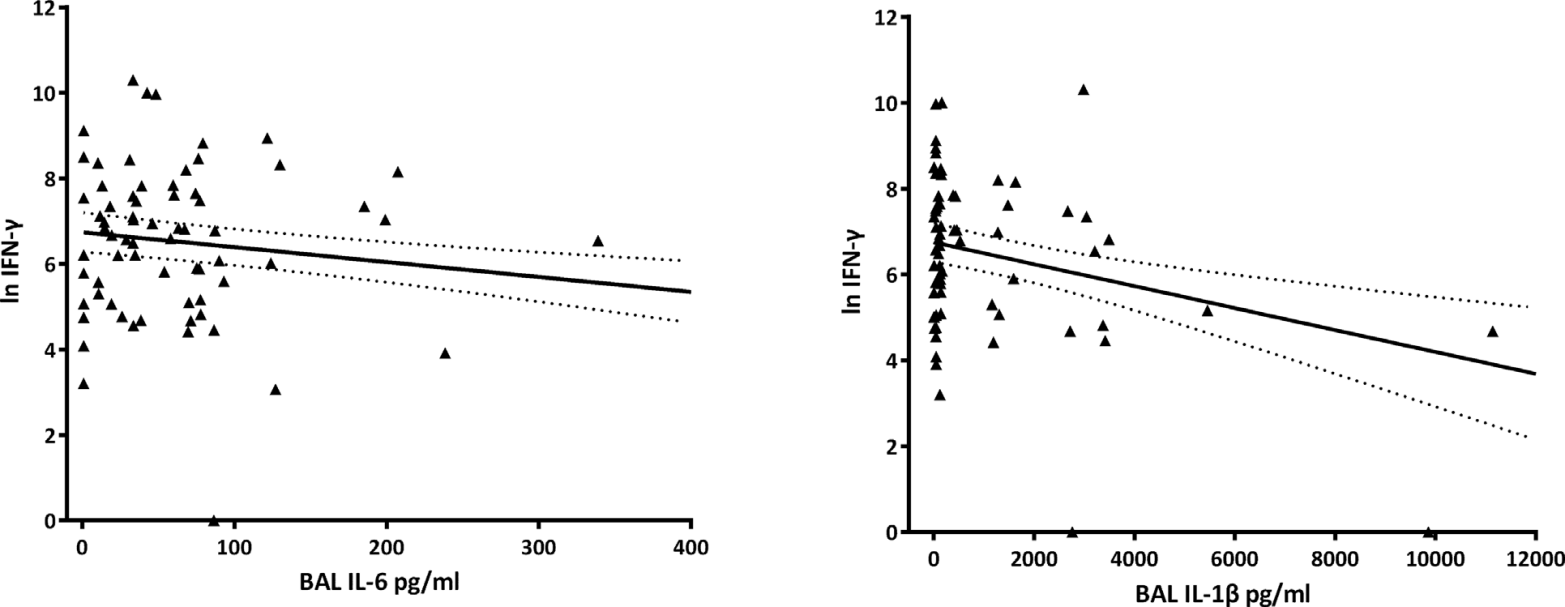
Shows the univariate regression model of NTHi-driven IFN-γ production by blood mononuclear cells and IL-1β a) and IL-6 b) concentration in the bronchoalveolar lavage fluid. As the multivariate analyses showed that only IL-1γ and IL-6 were independently associated with the capacity for NTHi-driven IFN-γ production, other BAL mediators are not shown in the figure.

On univariate analysis for the BAL data (Table 2 and Fig 1), % neutrophils, IL-1β, IL-6, IL-8, IP-10, NTHi infection and respiratory viruses were inversely associated with NTHi-specific IFN-γ production by blood mononuclear cells.

**Table 2:** Cellular, cytokine and microbiologic characteristics of bronchoalveolar lavage from children with CSLD and the association (univariate regression analysis) between the BAL markers of inflammation and NTHi-driven IFN-γ production by blood mononuclear cells.

BAL variable	median (IQR)	β	95% CI	p value
Total cell count x10^6^/ml,	0.36 (0.22–0.50)	-0.071	-0.222, 0.079	0.347
Neutrophil %	11 (4.6–39)	-0.023	-0.041, -0.005	0.015
Lymphocyte %	0 (0–0.7)	-0.081	-0.310, 0.149	0.485
Eosinophil %	2 (0.3–4.0)	-0.016	-0.122, 0.089	0.80
^#^ IFN-γ pg/ml	1.0 (1.0–16.9)	-0.228	-0.938; 0.481	0.52
^#^ IL-1β pg/ml	133.6 (44.6–1279.4)	-0.026	-0.039, -0.012	<0.001
^#^ IL-6 pg/ml	47.2 (19.3–77.8)	-0.349	-0.539, -0.158	0.001
^#^ IL-8 pg/ml	105.4 (30.5–547.7)	-0. 067	-0.141, 0.008	0.078
^#^ IL-12 p70 pg/ml	27.2 (1–63.6)	0.178	-0.401, 0.757	0.54
^#^ IP-10 pg/ml	340.7 (52.7–604.6)	-0.044	-0.067, -0.020	<0.001
^#^ LL-37 ng/ml	2.6 (1.9–4.2)	-0.275	-0.921, -0.371	0.40
Pos for any bacterial pathogen, n (%)	20 (28.1)	-0.2686	-1.298, 0.0761	0.60
*H*. *influenzae* (non-typeable)	11 (15.5)	-1.223	-2.4689, 0.022	0.054
Pos for any viral pathogen, n (%)	20 (29.4)	-0.5244	-1.564, 0.516	0.318
Rhinovirus	20 (29.4)	-0.5244	-1.564, 0.516	0.318

^#^ β and confidence intervals reported as 100x concentration.

On univariate analysis for the systemic inflammation data (Table 3), blood platelet levels (although within the normal clinical reference range) were inversely associated with NTHi-specific IFN-γ production by blood mononuclear cells. In contrast, blood leukocyte numbers, CRP and serum inflammatory mediators were not associated with NTHi-specific IFN-γ production.

**Table 3:** Cellular, cytokine and clinical characteristics of blood from children with CSLD and the association (univariate regression analysis) between the systemic markers of inflammation and NTHi-driven IFN-γ production by blood mononuclear cells.

Blood variable	median (IQR)	β	95% CI	p value
White cell count x10^9^/L	11.4 (9.1–14.3)	0.013	0.002, 0.029	0.087
Neutrophil	4.1 (2.85–5.55)	-0.064	-0.222, 0.095	0.42
Lymphocyte	4.9 (3.65–6.25)	-0.005	-0.143, 0.134	0.95
Eosinophil	0.9 (0.5–1.4)	0.311	-0.122, 0.744	0.16
CRP (n = 36) mg/L	1 (1–3.3)	-0.013	-0.061, 0.035	0.59
Protein g/L	73 (70–77)	0.009	-0.058, 0.075	0.80
Platelets x10^9^/L	337 (274.5–377.5)	-0.006	-0.010, -0.001	0.017
^#^IFN-γ pg/ml	26.6 (16.9–97.5)	0.074	-0.102, 0.251	0.40
^#^IL-1β pg/ml	144.4 (16.7–349.2)	-0.012	-0.089, 0.066	0.77
^#^IL-6 pg/ml	5.7 (1.0–19.4)	1.015	-0.621, 2.651	0.22
^#^IL-8 pg/ml	11.6 (1.0–17.8)	-0.111	-4.099, 3.876	0.96
^#^IL-12 p70 pg/ml	40.1 (15.3–206.5)	0.037	-0.127, 0.201	0.65
^#^IP-10 pg/ml	756.8 (506.6–1079.7)	-0.030	-0.135, 0.074	0.56
^#^LL-37 ng/ml	28.9 (22.0–38.3)	0.033	-0.404, 0.470	0.88

^#^ β and confidence intervals reported as 100x concentration.

### Multivariate analyses

Using a cut off of p<0.1 from the univariate analyses described in Tables 2 and 3 above and considering confounding factors, we combined the inflammatory markers in the BAL and blood into a multivariate linear regression model. We found that in vitro IFN-γ production by blood mononuclear cells in response to NTHi was significantly and inversely associated with BAL IL-1β (β = -0.023; 95%CI -0.04, -0.01; p = 0.001) and IL-6 (β = -0.316; 95%CI -0.49, -0.14; p = 0.001). In contrast, BAL IL-8, IP-10, neutrophil % and infection status, as well as blood platelets were no longer independent predictors of the IFN-γ response to NTHi by blood mononuclear cells

## Discussion

This study is the first to investigate the association between the systemic cell-mediated immune response and airway inflammation in children with CSLD. Our study of 70 children found that a reduced capacity for systemic NTHi-specific IFN-γ production was significantly associated with heightened airway inflammation as shown by high concentrations of IL-1β and IL-6 in BAL fluid. We found limited evidence of systemic inflammation in this cohort and neither markers of systemic inflammation or clinical markers of CSLD severity (including radiological scores of bronchiectasis and socio-demographic factors) predicted the capacity of the systemic NTHi-specific cell-mediated immune response. These data support the hypothesis that systemic adaptive immune responses are linked to persistent airway inflammation in children susceptible to lower respiratory infections.

The primary strength of our study is the inclusion of a relatively large number of young children with lung samples. There are few such published studies and none in children. Child-based studies are important, as in adult studies of chronic infection it is difficult to know if the measured immune responses are cause or effect. Our earlier study[8] documented a log-fold increase in IFN-γ production between healthy controls aged <18 months compared to those >18 months, consistent with normal maturation of the Th1 response through early childhood. However, in the CSLD group, we found a significantly lower capacity for IFN-γ production over the same age dynamic. Thus, early childhood studies, such as ours, may provide critical information regarding inflammation and the development of the adaptive immune response.

Indications of airway inflammation include changes in cellular profile and the presence of pro-inflammatory cytokines in the BAL or sputum. In adults and children with suppurative lung disease, airway inflammation is characterised by elevated levels of neutrophils, IL-1β, IL-6 and IL-8 [11,19,20]. Thus, we chose to focus on these cytokines. We found that, in our cohort of young children with CSLD, IL-1β, IL-6, IL-8 as well as IP-10 and LL-37 in the BAL were significantly correlated with BAL neutrophils. These markers of inflammation also negatively correlated with systemic NTHi-specific IFN-γ production on univariate analysis. However, on multivariate regression, only IL-6 and IL-1β were significantly and independently associated with NTHi-specific IFN-γ production.

The mechanism driving the relationship described is likely complex. The capacity for IFN-γ production was inversely associated with airway inflammation but the direction of this association (i.e. cause or effect) is unknown. One possible mechanism for the former involves regulating the transition between the inflammatory and adaptive response. These processes are integral for homeostasis through rapid identification and elimination of the invading pathogen and prompt resolution of inflammation, measured by markers such as IL-1β and IL-6.

IL1-β, produced by a variety of pulmonary cells in response to a microbial challenge, drives the inflammation cascade, localises neutrophils and promotes the production of inflammatory modulators, such as IL-6 and IP-10 production [21]. Consistent with this, we found a strong correlation between levels of BAL IL-1β, IL-6 and IP-10 in children with CSLD. IL-6 plays a complex role in the inflammatory response, from promoting inflammation to wound healing. Dysregulation of IL-6 is associated with chronic inflammation). In addition to its inflammatory modulating properties, IL-6 is integral to initiating the adaptive response and in directing its primary phenotype. In the lung, IL-6 polarises the adaptive immune response in favour of the humoral response. Animal and in vitro studies indicate this is accomplished in two ways. Firstly, dendritic cell-derived IL-6 suppresses activation of the Th1 pathway by inhibiting IL-12 production [22,23]. Secondly IL-6, in synergy with macrophage-derive IP-10, promotes the differentiation of B-cells into antibody-producing plasma cells [24]. Minimal numbers of lymphocytes in our BAL samples precluded our ability to study airway cells directly, however our data are consistent with the hypothesis that localised immune responses may not be contained to the lungs. We found that IL-1β, IL-6 and IP-10 were positively correlated with each other. Furthermore BAL IL-1β was positively associated with BAL IL-13. An environment high in these cytokines is conducive to a polarised humoral immune response. Thus it is plausible that the low systemic capacity for NTHi-specific IFN-γ production, also associated with BAL IL-1β and IL-6, may be a reflection of prolonged humoral responses in the lungs.

An alternative explanation for our finding regarding the association between increased local (BAL) inflammation and the poorer systemic adaptive response is that insufficient IFN-γ may contribute to increased susceptibility to infections with NTHi and subsequently a heightened state of local inflammation. There is increasing evidence to indicate that a strong systemic IFN-γ response is associated with protective immunity against NTHi [5,8] An impaired ability to produce sufficient IFN-γ in response to a challenge by NTHi may lead to an increased susceptibility to respiratory infections and prolonged inflammation due to ineffective clearing mechanisms. While the current cross-sectional study has found no relation between NTHi-specific IFN-γ production and severity of bronchiectasis, resolving this issue will require prospective studies examining the relationship between capacity for IFN-γ production and clinical outcomes.

We found limited evidence of systemic inflammation in our cohort of young children. There were isolated exceptions but overall, clinical markers of systemic inflammation including CRP, white cell count, neutrophils and platelets were within normal ranges. In contrast, adult based bronchiectasis studies have described systemic inflammation, although this may be transient and associated with bacterial infection [11,25]. We found no significant association between plasma and BAL IL-1β or IL-6 and no significant association between markers of systemic inflammation and the capacity for NTHi-specific IFN-γ production by blood mononuclear cells. Furthermore, clinical markers of CSLD severity (including radiological scores of bronchiectasis and socio-demographic factors) did not predict systemic NTHi-specific IFN-γ production. Thus, in children with CSLD, systemic NTHi-specific IFN-γ production is unlikely to be a direct consequence of systemic inflammation.

The cross-sectional design of the study meant we were unable to determine how resolution of airway inflammation affects the systemic NTHi-specific IFN-γ response. Also, minimal numbers of lymphocytes in our BAL samples precluded our ability to study airway cells directly. However our data shows a strong association between local inflammation and the systemic adaptive immune response to NTHi in children susceptible to lower respiratory infections. Future studies to identify the mechanisms driving this relationship between the adaptive IFN-γ response and airway inflammation are required to better inform effective, long-term prevention strategies for children at risk of CSLD. Understanding the nature of this association will help to determine if improving NTHi immunity would be best achieved by directly boosting the adaptive IFN-γ response through vaccination or immunotherapy strategies or if improved adaptive responses may be better realised by targeting IL-1β and IL-6 pathways in the lungs.

## Supporting Information

S1 Table Bacterial and viral pathogens identified in the BAL. (PDF)

S2 Table Spearman’s rank correlation of bronchoalveolar lavage neutrophils and eosinophils from bronchoalveolar lavage, with bronchoalveolar lavage markers of inflammation and infection. (PDF)
