# Supplementary material for: High Pulmonary Levels of IL-6 and IL-1β in Children with Chronic Suppurative Lung Disease Are Associated with Low Systemic IFN-γ Production in Response to Non-Typeable Haemophilus influenzae
Source: PLoS One. 2015 Jun 12;10(6):e0129517. doi: 10.1371/journal.pone.0129517 (PMC4466570; doi:10.1371/journal.pone.0129517)
Supplement: S1 Table — (PDF) [file pone.0129517.s001.pdf]

Supporting information

Table S1: Bacterial and viral pathogens identified in the BAL.

**Pathogen**	**Number of children (% of total N)**
Infection type identified	
Any (n=70)	41 (58.6)
[§]bacteria only	9 (13.2)
[§]virus only	21 (30.9)
[§]bacterial/viral co-infection	9 (13.2)
Any bacterial pathogen	20 (28.1)
H. influenzae (non-typeable)	11 (15.5)
S. pneumoniae	11 (15.5)
M. catharrhalis	3 (4.2)
K. pneumoniae	1 (1.4)
P. aeruginosa	3 (4.2)
C. pneumoniae	1 (1.5)
M. pneumoniae	0 (0)
Any viral pathogen (n=68)	30 (44.1)
Rhinovirus	20 (29.4)
Adenovirus	6 (8.8)
Enterovirus (n=35)	3 (4.4)
Bocavirus	3 (4.4)
RSV	2 (2.9)
Coronavirus	2 (2.9)
Wu	2 (2.9)
Ki	1 (1.5)
Parainfluenzae	1 (1.5)
Metapneumovirus	0 (0)

[§] n=68; 2 children were not tested for BAL viruses.
