# Supplementary material for: High Pulmonary Levels of IL-6 and IL-1β in Children with Chronic Suppurative Lung Disease Are Associated with Low Systemic IFN-γ Production in Response to Non-Typeable Haemophilus influenzae
Source: PLoS One. 2015 Jun 12;10(6):e0129517. doi: 10.1371/journal.pone.0129517 (PMC4466570; doi:10.1371/journal.pone.0129517)
Supplement: S2 Table — (PDF) [file pone.0129517.s002.pdf]

Supporting information

Table S2: Spearman's rank correlation of bronchoalveolar lavage neutrophils and eosinophils from bronchoalveolar lavage, with bronchoalveolar lavage markers of inflammation and infection.

**BAL**	**Neutrophils**		**Eosinophils**	
	**p value**	r_s	**p value**	r_s
IL-1β pg/ml	0.003	0.356	0.36	0.110
IL-6 pg/ml	<0.001	0.545	0.59	0.066
IL-8 pg/ml	<0.001	0.729	0.10	0.200
IP-10 pg/ml	<0.001	0.428	0.061	0.225
Eosinophils	0.059	0.227	-	-
Bacteria	0.51	0.081	0.31	0.124
Viruses	0.010	0.311	0.005	0.339
